# A prospective observational study of how veterinary clinics and their clients utilized a no-credit-check, third-party managed installment financing option to increase access to veterinary care

**DOI:** 10.3389/fvets.2025.1675999

**Published:** 2025-11-06

**Authors:** Heather J. Cammisa, Samantha Hill

**Affiliations:** 1 Open Door Veterinary Collective, Grand Rapids, OH, United States; 2 Independent Researcher, Geneva, OH, United States

**Keywords:** human–animal bond, veterinary care, access to veterinary care, veterinary relationship, payment options

## Abstract

The top client barrier to veterinary care is financial. Clients have reported their desire for more payment options, with recent research findings indicating that, with them, pet families could double the amount they could spend on lifesaving care. Research in 2022 reviewed cash and credit challenges that contribute to financial barriers and analyzed one option yet did not have direct engagement with clinics. This current study collected and analyzed data from 16 clinics to identify clinic and client impacts of expanded payment options in veterinary medicine. Clinics added at least one Varidi® payment option disassociated with a credit check of any kind. Clients reported why they used the payment option and the likelihood of alternatives they would have faced in the absence of having the option. Clinics overwhelmingly offered the option that guaranteed payment to the clinic. The average term was 9 months. The majority of those who used the option were existing clients of the clinic who sought sick, injury, surgery, or end-of-life care and received care at clinics offering credit-based financing. More than one in three cases (35.8%) faced a severe break in the human–animal bond (HAB), such as giving up their pet or putting their pet to sleep in the absence of the payment option. Combined with cases where the client was “very likely” to seek a lower cost option elsewhere, provide less care for their pet, or treat their pet on their own, 52% of cases met this risk to the clinic–client–patient relationship (REL), impacting clinic revenue and professional goals.

## 1 Introduction

More than one in four pet families report an inability to access needed veterinary care, with financial barriers reported as the top barrier to care (1). The cost for veterinary care is outpacing inflation (2). Through direct survey responses of clients and veterinarians, clients have been known to want opportunities to pay for veterinary care in installments rather than in full at the time of service since at least 2011 (3, 4). In a 2025 PetSmart study, 64% of pet families reported that they could double the amount of lifesaving care they could provide if interest-free pay-over-time options were available for 12 months (5). Nearly all of those with pets consider them family, with 51% saying they are as much family as humans. That figure rises to 64% among those with lower family incomes (6), with the benefits of the human–animal bond spanning the mental, emotional, and physical wellbeing of people (7, 8). In addition to incurred challenges, a lack of cash reserves among pet families across income brackets is associated with decreased perception of ability to access veterinary care (9). In 2022, two authors of this article published a retrospective observational study of 6 years of payment plan data collected by a third-party managed installment financing option without a hard credit check on clients to see how clinics benefited from using the tool (10). The dataset did not include definitive information about the clients’ motivations for using the tool nor the alternatives that clients would have faced in the absence of the tool. The dataset also did not have clinic user experience data or relationship measures for the client, clinic, and patient. This follow-up study was intended to gather information about how clients use a third-party managed pay-over-time option for financing the cost of care and the impact on veterinary clinics. To do this, the researchers recruited a convenience sample of veterinary clinics across the USA that agreed to offer a new financing option to their clients that did not require a credit check of any kind and to share data with the study group about the client accounts that they opened with this financial tool. This study worked with the financial tools offered by Varidi® because this provider agreed to add questions to their applications that would allow researchers to gather information from clients about why they were using this financial tool and what might have been the most likely outcomes for them and their pets if they had not had access to this financial tool. Researchers expected to find that when this type of installment plan was offered by veterinary clinics to pay for care, pets received care they might not have otherwise received; veterinary clinics got paid for services that might otherwise have been declined; and bonds between families, their pets, and their local veterinary clinic were maintained rather than broken.

## 2 Methodology

Advarra served as the Institutional Review Board (IRB) for this study, approving it as Exempted Pro00073759 and also approving a modification of the study with MOD02034102. Advarra IRB is registered with Office of Human Research Protection (OHRP) and Food and Drug Administration (FDA) under IRB#00000971.

### 2.1 Clinic participation

The study was promoted through social media, word of mouth, and personal contacts to seek clinics willing to offer Varidi® as a new financing option to their clients and to sign a data use agreement (DUA). Once the signed DUA was received, the clinic was enrolled in the study. Clinics were allowed to enroll any time from 1 October 2023 to 30 April 2024 and to open new client accounts until 30 September 2024. Researchers monitored client accounts through 31 March 2025 to allow for at least 6 months of data collection for any accounts that clinics opened later in the process.

Veterinary clinics were able to offer their clients two different financing options offered by Varidi, both of which do not perform any type of credit check on clients:

- Varidi guarantee plan (GP): Automatic coverage up to $4,000 is provided, with terms decided by the clinic. To qualify, clients’ monthly payments should not exceed 10% of their self-reported monthly income, and the clients should have a credit or debit card to make their monthly payments, are over 18 years of age, and have a valid form of identification. The tool provides a guarantee of payment to the clinic of the original amount in the original timeline in the event that a client does not pay the full amount or goes off schedule and needs to renegotiate their contract with Varidi. Clients pay an 18–22% one-time fee for the funds to Varidi at the time of account initiation. There are no other costs to the client for installments, interest, or penalties. The cost to the clinic is the merchant fee of 5% on client monthly payments and the time value of money in receiving payments over the term offered to the client. The minimum term is 6 months. Clients can repay earlier without penalty.^1^
- Varidi true payment plan (TPP): The amount of financing is at the discretion of the clinic. The client pays 5% up front to Varidi to initiate the payment plan. The clinic pays a 5% merchant fee on client payments. Varidi manages client payments but does not guarantee this type of payment plan if clients default.

Clinics had total control over whether they chose to offer GP or TPP options, to which clients, and under what terms; the use of other payment options at the clinic; and could alter how they used Varidi during the study. Clinics were compensated by the study covering a $59 monthly fee to effect a 5% merchant cost (otherwise 7% at the time of the study) and a one-time stipend of $100 to $250 for a staff training “pizza party.” Clinics were also offered protection against defaults from study funds if they used TPP up to a total limit of $5,049 to $25,244, depending on the size of the clinic.

The 16 participating clinics were emailed a post-study survey designed to investigate their user experiences and the criteria they used to offer the two financing options to their clients.

### 2.2 Client participation

The study team did not interact directly with any clients. The study team analyzed de-identified data Varidi collected from enrolled clinics and clients during their usual online application and payment management process, such as the type of care provided, self-reported household monthly income, homeownership status, employment tenure, terms of the contract, frequency of visits with the clinic, and payment performance data.

For this study, Varidi also added two new questions to their application to investigate the clients’ reasons for applying for a financing option and what their outcome(s) would most likely be if they could not use the option. However, Varidi did not participate in analyzing the collected data or reviewing the results presented in this study.

The new questions added to the application were:

1.  Why did you not use a hard credit type of financing such as CareCredit®, Scratchpay®, Wells Fargo®, and iCare Financial®. to finance your pet’s current veterinary needs?

Answer options:

- I was not approved.
- I was only approved for part of the amount.
- I did not want to have a hard credit check run.
- This option was not presented to me.
- I do not want to answer.

2.  Without access to this payment plan, how would you rate the following options for your veterinary needs? (very likely, somewhat likely, likely, not at all likely, and neutral)

Answer options:

- Provide less care for my pet at this clinic.
- Find a lower-cost option elsewhere.
- Surrender my pet to an animal shelter.
- Give my pet to someone else.
- Treat my pet on my own or wait and see how it goes.
- Put my animal to sleep.
- Seek funds from friends or family.
- Apply for and seek to open another credit card.

## 3 Results

### 3.1 Clinic data

Due to the open methods used to recruit clinics to enroll in the study, it is unknown how many clinics were aware of the opportunity. A total of 45 clinics were directly engaged following promotion of the study through contact with the researchers, 20 of them signed the DUA to enroll in the study, and 17 clinics participated by initiating at least one client account through Varidi (37.8% of participation rate). One of the 17 participating clinics was lost to follow-up, and their data were removed from the study. Overall, 16 clinics remained in the data for analysis: 12 for-profit clinics (9 clinics identified as general practice, 2 identified as combined general, specialty, and emergency, and 1 identified as emergency) and 4 non-profit clinics (3 clinics identified as general practice and 1 identified as combined general, specialty, and emergency).

A total of 424 client accounts were opened during the study that provided care for a total of 444 animals (or cases). The 16 clinics had full-time equivalent (FTE) veterinarians ranging from 1 to 5.5 FTE, with a median of 2.0 veterinarians. The median annual revenue for all of the clinics was $1.4 million.

Of the participating clinics, the 4 non-profits were the top users of the payment options by initiating 73.1% (310 of the 424) of the client accounts for 74.3% of the cases (330 of the 444 animals), accounting for 62.8% of the purchased veterinary care ($166,146.42 of $264,476.65 of total veterinary care with Varidi). The non-profit clinics reported that their clients were either predominantly low-income (two clinics) or economically diverse (two clinics). The median monthly income directly reported by these clients was $2,500 ($30,000 annually). Three of the non-profit clinics strongly agreed or agreed with the statement, “Payment options like Varidi will enable us to expand the care that we can provide to clients in financial need, thereby expanding the care that clients can access.”

The 12 participating for-profit practices (Emergency (ER), combined, and general Practice) accounted for 26.9% (114 of the 424) of the client accounts and reported that their clients predominantly fell under middle-income (seven clinics), economically diverse (three clinics), and low-income (two clinics) categories. The median monthly income directly reported by these clients was $3,500 ($42,000 annually). On a case-count basis, for-profits provided care for 25.7% (114 of the 444) of animals. Nine of the for-profit clinics (75%) strongly agreed or agreed with the statement, *“Payment options like* Var*idi will enable us to expand the care that we can provide to clients in financial need, thereby expanding the care that clients can access.”*

Despite the study pledging to guarantee Varidi’s TPP option for clinics, the clinics demonstrated a strong preference for the GP option (94.6% of client accounts, 401 of 424) over the TPP option (5.4% of client accounts, 23 of 424). In total, 14 of the participating clinics indicated that the payment guarantee was “very important,” 1 clinic responded “neutral,” and 1 clinic did not answer the question. Other response options were “important” and “not important.”

Nearly all clinics in the study (87.5%, 14 of 16) offered additional credit-based financing options during the study period (i.e., CareCredit® or ScratchPay with either 6-month or 12-month terms). More than half of the clinics (56.3%, 9 of 16) reported offering Varidi only after their clients were declined by another credit-based financing option or lacked other payment options. Some reserved Varidi for their existing clients only or offered it based on the total cost of care. Some clinics changed their criteria for offering Varidi during the study and so offered it in multiple ways.

The mean average repayment term offered to clients was 9 months across all client accounts (Table 1).

**Table 1:** Accounts and veterinary care amounts by clinic type.

Clinic type	Amount of veterinary care purchased using GP	Amount of veterinary care purchased using TPP	Total accounts and veterinary care dollars
Non-profit	$165,237.55	$908.87	310 (98.7% GP/ 1.3% TPP) $166,146.42 = total amount of veterinary care purchased
For-profit	$82,281.32	$16,048.91	114 (83.3% GP/ 16.7% TPP) $98,330.23 = total amount of veterinary care purchased
Total	$247,518.87	$16,957.78	424 (94.6% GP/ 5.4% TPP) $264,476.65 = total amount of veterinary care purchased

### 3.2 Client data

Details about client income, employment, homeownership, and history with the practice were only collected for GP accounts during the application process.

Clients who applied for the GP option reported:

- They were existing clients of the practices (76.1%), who had 1–3 prior visits with the clinic (43.7%) or 4 + prior visits with the clinic (32.4%).
- 23.9% were new clients of the clinic.
- Median monthly household income of $2,500, equating to $30,000 annually.
- The majority were non-homeowners (63.3%).

Overall, the clients used the Varidi options to obtain $264,476.65 worth of veterinary care at the 16 participating clinics. The accounts were opened for median treatment costs of $498.85 and average treatment costs of $623.77, with median monthly payments of $60.00 and average monthly payments of $64.74.

When applying for a new account, clients were asked why they were using the payment option. A total of 216 clients either did not provide a response or said, “I do not want to answer” to the question. Of the clients who did indicate why they were applying for the payment option (*n* = 208), 39.9% said it was because they had either been turned down or did not qualify for the full amount of treatment by another payment option available. Another 48.1% indicated that they did not want to have a hard credit check run. Finally, 12.0% indicated that the opportunity was not presented to them to apply for other financing for their pet’s care.

The majority of the clients (93.3%) answered the multi-part question on the Varidi application about what their outcomes would be without opening an account, with a minimum of one-third of all clients indicating “very likely” for each of the options, by account:

- Provide less care for my pet at this clinic (37.7% of all clients)
- Find a lower cost option elsewhere (42.2% of all clients)
- Surrender my pet to an animal shelter (34.0% of all clients)
- Give my pet to someone else (33.3% of all clients)
- Treat my pet on my own or wait and see how it goes (38.7% of all clients)
- Put my animal to sleep (34.0% of all clients)

The researchers analyzed negative risks to the human–animal bond and the veterinary-client-patient relationship using “very likely” responses to outcomes if the payment plan was not available as follows, per case:

- Risk 1: human–animal bond (HAB): The risk of breaking the human–animal bond. “Very likely” to any of the following questions: “give my pet to someone else,” “surrender my pet to an animal shelter,” or “put my animal to sleep.”
- Risk 2: clinic–client–patient relationship (REL): The risk of breaking the client–patient relationship with the veterinary clinic and losing potential current and future revenue. “Very likely” to “provide less care for my pet,” “find a lower-cost option elsewhere,” “treat my pet on my own,” and/or being at risk of HAB (Table 2).

**Table 2:** Description of cases by risk and clinic type.

Risk type	Risk type by clinic type	% of total cases (444)	Average treatment cost per case	Average term per case (months)	Average monthly payment per case	Median HH income
Human–animal bond, overall (*n* = 159)		35.8%	$613.19	10	$59.55	$2,500
	At high risk of breaking HAB at non-profit clinics (*n* = 134)	30.2% of total cases 40.6% of nonprofit cases	$543.50	10	$51.36	$2,500
	At high risk of breaking HAB at for-profit clinics (*n* = 25)	5.6% of total cases 21.9% of for-profit cases	$986.75	9	$103.42	$3,720
Relationship risk, overall (*n* = 231)		52.0%	$571.93	9	$58.32	$2,500
	At high risk of breaking relationship with non-profit clinic (*n* = 195)	43.9% of total cases 59.1% of non-profit cases	$492.03	9	$49.19	$2,375
	At high risk of breaking relationship with for-profit clinics (*n* = 36).	8.1% of total cases 31.6% of for-profit cases	$1,004.77	9	$107.78	$4,000

The researchers recoded clinic-reported care provided into treatment types (e.g., sick) and also by the urgency of the care needed (e.g., emergency). If the account was opened for more than one care plan, the researchers selected the less discretionary, higher-level care for analysis. If the type of care or urgency could not be discerned by the researchers, it was coded as “unknown.” Because some client accounts were opened for care for more than one pet (e.g., three puppies being treated for parvovirus), the researchers analyzed the data by both individual cases (animals served) and by client payment accounts (Table 3).

**Table 3:** Description of cases by case type and case urgency.

Case type	Case urgency	% of total cases (444)	Average treatment cost per case	Average term per case (months)	Average monthly payment per case	Median HH income
Total sick, injury, surgery, or end of life (*n* = 295)		66.4%	$694.85	10	$68.07	$2,500
	Urgent or emergency sick, injury, surgery, or end of life (*n* = 79)	17.8%	$986.21	10	$86.85	$3,000
	Non-urgent or non-emergency sick, injury, surgery, or end of life (*n* = 216)	48.6%	$588.28	10	$61.20	$2,417
Wellness, dental, altering (*n* = 131)		29.5%	$369.83	7	$45.56	$2,085
Unknown (*n* = 18)		4.1%	$613.83	8	$77.76	$5,000

Seven in 10 of HAB cases (69.2%) and six in 10 of REL cases (61.5%) involved treatment for sick, injury, surgery, or end-of-life care.

For a basic measure of clients’ ability to pay for expanded options, researchers looked at accounts when (a) Varidi had stepped in to pay clinics for all or part of the payments (GP), (b) when the account was delinquent at the end of the study data window, or (c) when clinics had not engaged the guarantee (TPP or GP). The latter included some clinic errors in usage. Of the total cost of services provided, 13.5% is not fully paid via original terms by the client using this breakdown. However, due to the GP providing clinics with payments on behalf of clients that were approved and defaulted on payments, only 4.6% of the total cost of services was not paid to the clinics ($12,263.61). This lost revenue includes default amounts on TPP accounts ($1,575.88) and GP accounts where the clinic did not process the request for Varidi to take over payments of the account or did not process the guarantee application correctly ($10,687.73).

## 4 Discussion

The findings in this study support offering alternative payment options disassociated with a credit check to increase access to veterinary care for the benefit of people, patients, and practices. These data described in this study suggest that by offering—and client usage of—a guaranteed, no-credit-check, third-party-managed pay-over-time option, there are benefits in maintaining relationships with clients, providing care needed to patients, and increasing practice revenue. Among cases where human–animal bond-preserving care was achieved, the average cost of treatment was $613.19 per case, with clients being able to pay over an average of 10 months through average monthly installments of $59.55 per month. This alternative payment tool provided an option for clients who were turned down for credit by other financing options or who did not want to have a hard credit check appear on their credit reports (or, perhaps, knew they would not qualify) to pay for their pet’s veterinary care. As a result, the 16 veterinary clinics that participated in this study added more than a quarter of a million dollars to their revenue that may otherwise have been lost, a very likely outcome for the 52.0% of cases found to face a high relationship risk with the clinic for the client and their pet in the absence of the payment option.

Because the financial option offered in this study allowed each clinic to set terms as needed for their clients, it is important to note that on average, the clinics chose to set terms of 9 months for their clients to pay their account balances. Commonly used credit-based tools in the veterinary industry can charge clinics higher merchant fees after the 6-month term point, and clinics may be less likely to offer these terms that are needed by clients to effect monthly payment amounts that they can afford, if clients qualify for them.

Using the 12-month treasury bill as a discount rate to give a present value comparison to “up-front” payments a clinic may receive from credit-based (or any other) financing options that pay clinics within a few business days, the “cost” of receiving funds over the term of the average Varidi plan (using $65 per month per account for 9 months) is $11.02 for the clinic, or 1.9% of the initial amount. Adding that to the merchant cost brings the total clinic cost on the average account to 6.9% for comparison to merchant costs of other options in the marketplace, noting that clients may not qualify for those other options.^2^

Clinics can compare their costs and client realities (costs and access) of different options to prepare their own financial triage plan for meeting clients with payment options that balance clinic and client needs.

### 4.1 Client impacts

The human–animal bond is at severe risk for approximately 4 in 10 cases (35.8%), reporting that clients were “very likely” to give up their pet to a shelter or another person or put their pet to sleep in the absence of the Varidi payment option offered to them. This finding, across combined clinics and treatments, supports the hypothesis that expanded payment options disassociated with credit checks offer the ability to avert serious breaks in the human–animal bond by enabling clients to access veterinary care. Pet families most likely to lose their relationship with their pet were able to access bond-preserving care for payments of $59.55 per month, on average. The finding for averting pet surrender (34% of accounts reported “very likely” to this direct question) is especially salient for the animal welfare community, as two of three pet owners who surrendered their pets to a shelter felt that resources, such as access to affordable/free veterinary care (25%) and financial support for medical issues (20%), could have prevented the surrender (11).

The average term of 9 months offered to clients indicates that the veterinary field should offer payment options with terms beyond 6 months to meet clients with a monthly payment that they can afford. Clients showed a reservation in disclosing why they were using the payment option; although among those who did respond, the majority of them indicated that they were either turned down, did not qualify for the treatment amount, or did not want to have a hard credit check run. In fact, 98.4%, or 437, of the cases that used Varidi were with clinics that offered alternative credit-based financing.

While the majority of payment options offered to clients were the ones with a guarantee of payment to the clinic, payment performance data showed that clients were able to pay these bills at a high rate (86.5%, conservatively, of treatment cost), pointing to clinics and clients using these tools well, on the whole. To provide a basic client cost of funds comparison, if clients had room to pay for treatment on a credit card offered at the published interest available in Q2 2024, they would pay approximately $49.45 in interest on a $585.00 bill if they could only pay $65 per month, as in the example above.^3^ Varidi’s cost of funds for clients is higher, at $105.30 to $128.70 to set up the payment account (18–22% fee). Varidi does not have interest, installment payments, or penalties, which are additional factors to evaluate between options for clients and clinics.

Clients were seen to mostly secure treatment for sick or injured pets or surgeries using the payment option, although some clients were able to access wellness care, altering procedures and dentals.

## 5 Conclusion

Parties that seek to support the human–animal bond with veterinary care, from veterinary professionals to animal welfare advocates to social service agencies, should view financing options, notably those disassociated with a credit check and offered beyond 6-month terms, as paths to mitigating financial challenges of pet families in obtaining veterinary care. Payment vendors in this endeavor should be viewed as key collaborators, and open discussions between the industries should occur to bring those solutions forward, in use, and for wide awareness of availability. Clients have been asking for expanded options. Clinics can retain existing clients and acquire new clients who face financial challenges unmet by credit-based tools while meeting professional goals of caring for animals.

### 5.1 Areas for further research

Further analysis of clinic experiences from the survey will offer more texture as to why clinics opted for the guarantee plan strongly over the non-guarantee plan and what their user experience was in using the payment option, as well as their desires for options in the industry.

Additional research is needed to capture client experiences, full pet family and socioeconomic dynamics, and payment option preferences.

Additional research is needed on combined strategies of spectrum of care, payment options, angel funds, and/or donor funds to meet clients who cannot afford the full treatment cost even with payment options. Further investigation can identify what clinics need to effectively and efficiently implement payment triage for clients, addressing key steps, training, and elements for success.

### 5.2 Limitations

This type of study design has limitations because it does not account for clinics that choose to participate. There is no control group for comparison, and clinics used the tools as they felt best met their clinic and client needs. Therefore, usage was not homogenous. The clinic sample size is modest, and case data are skewed to non-profits, as they used the payment tools at a higher level than for-profit clinics. Given the client income levels at the non-profits, the results are biased to those income-range clients.

Researchers found that the state of the veterinary industry presented a challenge in onboarding clinics to the study in 2023 and 2024 related to staff shortages, turnover, and burnout.

## Data Availability

The raw data supporting the conclusions of this article will be made available by the authors, without undue reservation.

## Footnotes

^1^At the time of the study, the merchant fee would have been 7% for non-study clinics unless clinics paid a monthly fee of $59 to effect a 5% merchant fee. The break-even point for the fee was, therefore, $59/0.02 = $2,950 per month in care. Hoping that study participants would use the tools at this level, the study paid their monthly fee to effect the 5% rate. The rate applies to both GP and TPP options. Varidi has since eliminated the monthly fee and now charges a merchant fee of 6% to all clinics, which did not impact the study.

^2^PV = PMT * [1 − (1 + *r*)^-*n*]/*r*, where PV = Present value of pay-over-time payments to clinic; Pmt = The average monthly payment made by clients less the 5% merchant cost to clinic; *r* = The 1-year treasury bill rate at 30 April 2024 on a monthly basis, 4.83%/12; *n* = The term, 9 months.

^3^Published rate 22.78% at Q2 2024. Credit card interest rate where interest charged. Assumed first month no interest. https://www.federalreserve.gov/releases/g19/current/
